# “The significance of clinical foetal autopsy for reproductive health care: an ethical analysis in the German context”

**DOI:** 10.1007/s11019-025-10265-8

**Published:** 2025-04-03

**Authors:** Elisa Groff, Marta C Cohen, Florian Steger

**Affiliations:** 1 Institute of the History, Philosophy and Ethics of Medicine, University Hospital Ulm, Ulm, Germany; 2 Histopathology Department, Sheffield Children’S Hospital NHS, Sheffield, UK

**Keywords:** Perinatal mortality, Clinical foetal autopsy, Reproductive medicine, Fertility preservation, Medical ethics, Health care

## Abstract

The clinical autopsy of foetuses, stillborn babies, or neonates and the examination of the placenta help to inform the mother’s decision-making and her medical care for subsequent pregnancies. Indeed, these post-mortem examinations can identify unexpected congenital malformations or the cause of repeated miscarriage or stillbirth. However, the use of clinical pathology for diagnostic purposes in the context of family planning for bereaved parents with an unfulfilled desire to have a child, and IVF couples has received little attention to date. This article applies Beauchamp and Childress’ bioethical framework to identify, assess and systematically discuss ethical issues associated with the use of clinical foetal autopsy in reproductive healthcare within the German legal context. In the format of a clinical ethics consultation, the article examines the current policy on perinatal post-mortem examinations in Germany, and asks whether the clinical foetal autopsy for reproductive health purposes should be part of standard clinical examinations offered to bereaved parents. The conclusion of our research recommends clinical foetal autopsy as ethically acceptable, provided that the necessary resources are available. This recommendation is based on the ethical obligation towards the mother as the patient, which is grounded in the bioethical principles of autonomy and beneficence.

## Background

After a miscarriage, stillbirth, or the loss of a newborn baby, clinical autopsy and the examination of the placenta can provide crucial information to the family and to the reproductive medicine team (Doughty et al. 2024; Gallagher et al. 2023; Redline et al. 2022; Sarioglu and Turowski 2015). These key insights can inform the mother’s decision-making and her medical care for subsequent pregnancies (Loverro et al. 2022; Rossi and Prefumo 2017; Godbole et al. 2014). Indeed, the results of post-mortem examinations can be used to advise bereaved parents with an unfulfilled desire to have a child and IVF couples, as they help to clarify unexpected congenital malformations, genetic conditions or the cause of repeated miscarriage, stillbirth or perinatal death (Ernst 2014). This, in turn, can help to reduce the risks for future pregnancies (Elayedatt et al. 2023; Cassidy et al. 2019). Accordingly, close cooperation between obstetrics, neonatology, perinatal pathology, human genetics and reproductive medicine is necessary in order to improve the medical care of the mother and future offspring. This requires that case-related relevant findings are shared and discussed, preferentially through multidisciplinary team meetings. This, in turn, raises fundamental ethical questions of data protection and data sharing permission (Ventura Hernández et al. 2022; Cassidy et al. 2019; Tennstedt 2002). Furthermore, it urges to educate the public about post-mortem procedure and about the potential benefits a clinical foetal autopsy brings with it in the context of fertility preservation and family planning (Pakanen et al. 2022; Ramos et al. 2021; Gordijn et al. 2006). Informed consent underpins the clinical autopsy pathway, and this should be transparent and comprehensive in order to enable bereaved parents or reproductive partners to make autonomous reproductive choices according to their preferences (Groff and Steger forthcoming; Rugge et al. 2021; Lane 2016). Closely related to post-mortem decision-making is also the ethical question whether medical professionals are adequately competent to navigate the process of informed consent after the death of a fetus or of a baby in the hospital (Reed et al. 2021; Amoroso 2016).

Autopsy practice accommodates not only legal regulations but also ethical and cultural expectations (Rugge et al. 2021; Kaiser 2010; Gordijn et al. 2006; Lynch 2002: p. 69). Protocols governing informed consent prior to autopsy and the use of post-mortem samples and findings not only vary internationally, but also from regions in the same country, and locally from hospital to hospital (Alfsen et al. 2022; Ventura Hernández et al. 2022). Similarly, hospital services for the mother following a perinatal loss are not uniformly regulated in the healthcare sector in Europe (Kalanlar 2020; Sereshti et al. 2016; Šmídová 2022). In Italy, for example, foetal autopsy is mandatory for diagnostic purposes following a miscarriage or a voluntary termination of pregnancy, according to the Prime Minister’s Decree dated 9 July 1999. Since 2006, also cases of sudden foetal and infant death are covered by the law and are subject to mandatory post-mortem examination (Bonsignore et al. 2023). The implementation of such a regulation helps to engender the establishment of the requisite conditions for the attainment of self-determination on the part of the mother. In contrast, post-mortem examinations in Germany are not regulated by a single binding law (Research Services of the German Bundestag 2016, 3–3000−201/14; Dettmeyer und Madea 2004). Furthermore, the mother’s decision to perform autopsies on neonates and perinatal cases is not uniformly codified in German hospital admission agreements (Dettmeyer und Madea 2002; Dettmeyer und Madea 2004; Hörnle 2010: pp. 118–122). Although there are recommendations on how to handle stillbirths, miscarriages and neonatal deaths in clinical settings by the German Hospital Association, the Research Services of the German Bundestag, and some archdioceses for Catholic-run hospitals, these only refer to the various burial and civil status laws (Research Services of the German Bundestag 2016, 3–3000−201/14).

The Federal Association of German Pathologists’ working group on autopsies has compiled a list of indications for conducting clinical post-mortem examination (Friemann 2014). According to this list of recommended actions, the examination of the placenta and clinical autopsy should be performed as standard in cases of stillbirths, miscarriages, neonatal and perinatal deaths to determine the cause of death. Prior to this, bereaved parents or reproductive partners should receive individual medical and psychological counselling (Federal Association of German Pathologists’ Guidelines 2017:p. 4, 11). This list of indications considers the results of a clinical autopsy as providing critical insights into the mother’s decision-making and her future medical care for subsequent pregnancies. Despite the evidence, clinical foetal autopsy remains only a recommended action in the German healthcare sector though. Neither the obstetricial nor the neonatologist has a legal obligation to offer an autopsy following neonatal or perinatal death (Tennstedt 2002). Even the “Statement on Autopsy” released by the German Medical Association in 2005 does not address the significant connection between foetal pathology, reproductive medicine and sexual and reproductive health.

Strikingly, even associations of bereaved parents and volunteer-based organisations in Germany, which are committed to raising public awareness of death at the beginning of life through the creation and dissemination of pertinent and updated material, refrain from making stronger demands on recommending foetal autopsy under relevant conditions.^1^

In summary, while few international studies have explored the use of clinical pathology for diagnostic purposes (Doughty et al. 2024; Elayedatt et al. 2023; Sarioglu and Turowski 2015; Cassidy et al. 2019), there has not yet been a systematic analysis of the ethical issues associated with clinical foetal autopsy for reproductive health purposes (see e.g. Rugge et al. 2021; The Australian National Code of Ethical Autopsy Practice 2018).

Therefore, this study aims to identify, assess and systematically discuss ethical issues associated with the use of clinical foetal autopsy in reproductive healthcare within the German legal context and according to Beauchamp and Childress’ four bioethical principles of “autonomy”, “beneficence”, “non-maleficence” and “justice” (Beauchamp and Childress 2019). By applying the format of a clinical ethics consultation (Orr and Shelton 2009; Steger 2013), this research examines the current German policy on foetal, neonatal and perinatal autopsy, and asks whether—from an ethical standpoint—the offer of a clinical foetal autopsy for reproductive health purposes should be part of standard clinical examinations. Provided that the necessary resources are available, and thus the bioethical principle of justice is respected, the research recommends clinical foetal autopsy as ethically permissible. This recommendation is based on the ethical obligation towards the mother as the patient, which is grounded in the bioethical principles of autonomy and beneficence (Groff and Steger forthcoming; Chervenak and McCullough 2021).

## Method

In the first stage of our analysis, we examine the clinical autopsy of foetuses, stillborn and neonates in the context of family planning by applying Beauchamp and Childress’ four bioethical principles. The analysis is conducted within the framework of the mother’s rights as a patient in general, and in particular, her right to reproductive self-determination. In this context, the question arises as to what extent the clinical autopsy of the foetus serves to determine the cause of death and the quality of medical care, or if it also serves the reproductive healthcare of the mother with regard to future pregnancies.

In the second stage of our analysis, we discuss whether performing a clinical foetal autopsy for reproductive health purposes constitutes an ethically illegitimate instrumentalization of the foetus (Knoepffler 2021: pp. 156–157; Groß et al. 2010: pp. 350 − 51; Hoerster 2002: p. 15; Beyleveld und Brownsword 2004: p. 30). Ultimately, we make a recommendation for the healthcare professionals, which is appropriate to present health care standards and based on the following ethical aspects: the mother’s preferences, the mother’s time-limited fertility, her quality of life, the mother’s and her reproductive partner’s personal values, and contextual features (Orr and Shelton 2009).

### Ethics question

The present analysis, which is framed as an ethics consultation, addresses the following ethical question: Should the offer of a clinical foetal autopsy for reproductive health purposes be part of standard clinical examinations?

From an ethical standpoint, there are three arguments that could be made against conducting autopsies on foetuses, stillborn and neonates for reproductive health purposes:

1. The primary purpose of clinical foetal autopsy should not be to serve as a preventive measure to avoid future pregnancy risks.
2. The primary purpose of clinical foetal autopsy should not be to serve as a medical indication for fertility preservation.
3. The use of clinical foetal autopsy for reproductive health purposes is unethical, as it instrumentalises foetuses, stillbirth and neonates to serve the mother’s unfulfilled desire to have a child.

### The primary purpose of clinical foetal autopsy should not be to serve as a preventive measure to avoid future pregnancy risks (1)

In principle, the mother can request an autopsy after foetal, neonatal and perinatal loss as part of her right to self-determination and her right to receive information as a patient (Davies 2020). Indeed, the results of post-mortem examinations can clarify the cause of repeated miscarriages or stillbirths and, on this basis, be used as a medical indication for preventive treatment to reduce the risks in future pregnancies (Elayedatt et al. 2023; Loverro et al. 2022; Cassidy et al. 2019; Rossi and Prefumo 2017; Godbole et al. 2014). This therapeutic goal is in line with the European Charter of Patients’ Rights, which states that every individual has the right to “preventive measures” (art. 1) and to access to healthcare (art. 2) https://ec.europa.eu/health/ph_overview/co_operation/mobility/docs/health_services_co108_en.pdf Moreover, according to Beyleveld’s and Brownsword’s concept model of preserving human dignity in elective medical treatments (2004: pp. 231 − 32), clinical foetal autopsy is ethically and legally acceptable. Indeed, the mother’s request for an elective post-mortem examination is not driven by fetishism, nor does it negate the rights of the foetus. However, maternal preference for requesting a clinical autopsy after foetal, neonatal and perinatal loss is contingent on (1) the availability of resources (2), the legal context in which the mother is based, and (3) the awareness of the mother and her reproductive partner of the possibility of a foetal autopsy. This means that autopsy as an elective measure to improve the mother’s quality of life—in terms of fulfilling her wish of future pregnancies—is limited to the provision and equal distribution of resources within the healthcare system in which the mother is insured. This also refers to the right to access healthcare without discrimination (McLean 2000; Groff and Steger 2024) and corresponds to the fourth bioethical principle of “justice”.

### The primary purpose of clinical foetal autopsy should not be to serve as a medical indication for fertility preservation (2)

Considering the aforementioned assessments, the mother’s right to develop her own personality (Art. 2 of the Basic Law for the Federal Republic of Germany) and “freedom to write her life story“(Barak 2015: pp. xix-xx), the issue arises whether the medical history of the mother holds equal importance to her future life plan. In other words: is it ethical for the healthcare system to refrain from using available resources, such as clinical autopsy, to potentially improve the health of the bereaved mother in terms of fertility chances?

To untangle this ethical issue, we further analyse the circumstances of a clinical foetal autopsy from the perspective of the mother, and in relation to the second and third bioethical principles of “beneficence” and “non-maleficence” by discussing the mother’s preferences, quality of life, and contextual features (Orr and Shelton 2009; Porz 2008).

The initial step in a clinical ethics consultation is to inquire about the patient’s preferences, as each individual establishes the framework and boundaries of their values as the basis for their healthcare decisions (Porz 2008). Therefore, assessing the patient’s ability to maintain their standard of living (Killmister 2010; Porz 2008: pp. 154–160) and to achieve their life goals is a central element of patient-centred care (Kwame and Petrucka 2021; Riedel et al. 2023), which is respectful to global strategies on current health services (WHO 2019). In the context of a perinatal death in the hospital, the mother’s request for a post-mortem examination is based on her personal values (Lipp 2019: pp. 35–36) and on her “immediate possibility of action” (Killmister 2010:1p. 62), i.e. her past experiences in terms of medical history, her time-limited fertility, and her family planning (Porz 2008: pp. 125–135, 153–173). All of these factors affect the mother’s ability to act and make autonomous decisions in the present, i.e. in the aftermath of perinatal loss. Therefore, providing the option of a clinical foetal autopsy as part of the mother’s healthcare planning can have a dual effect on the mother’s physical and mental health (Ernst 2014). Firstly, it strengthens her resilience by providing knowledge of immediate available healthcare resources, which informs her decision-making and her medical care both in the present and in the future (Porz 2008: pp. 156–158). Secondly, it restores her capacity for action – in terms of her “latent potential” (Killmister 2010), which was neutralised by the death of her baby (Porz 2008: p. 153). In sum, maternal preference for having a clinical autopsy following miscarriage, stillbirth or neonatal loss is an ethical indication for recommended action in maternal health care, one that is appropriate to current health care standards. Indeed, granting the bereaved mother the ability to have an elective clinical autopsy, after a conversation with the physician addressing the scope and goals of this procedure, can empower her during the bereavement process and in subsequent pregnancies (Westgate et al. 2023; Reed et al. 2021; WHO 2019; Hall et al. 2017; Garthus-Niegel et al. 2014).

### Clinical foetal autopsy for reproductive health purposes instrumentalises foetuses, stillborn and neonates (3)

What follows is an analysis of potential ethical conflicts between the mother patient’s rights and the rights of the foetus. The discussion revolves around whether ethically, clinical autopsy of foetuses, stillborn and neonates for reproductive health purposes instrumentalises the deceased (Davis 2020; Beyleveld und Brownsword 2004: p. 30; Human Genetic Advisory Commission and Human Fertilisation and Embryology Authority 1998:§ 8). The dignity of the deceased is compared here to that of potential future child. The following question guides the discussion: to what extent can deceased foetuses, stillborn and neonates be “used” for the benefit of the mother’s health and future pregnancies without instrumentalising their dignity? This leads to the question of how the dignity of foetuses, stillborn and neonates is defined in the given legal context.

In Germany, the ontological and legal status of babies who died in the womb, stillborn babies, or babies who died in childbirth is regulated in the Personal Status Law (19.02.2007: BGBl. I S. 122; 17.07.2023: BGBl. 2023 I, nr. 190, §§ 21, 31) and Burial Law. According to Section 31(1) of the German Personal Status Law, a “live birth”, *Lebendgeburt*, has taken place if after separation from the mother’s womb, either the heart beats or the umbilical cord pulsates or natural pulmonary respiration has begun. According to Section 21(2), a “stillbirth”, *Totgeburt*, has occurred if (1) none of the life signs described for a live birth are detectable, (2) the weight of the baby is at least 500 g, or (3) the weight of the baby is less than 500 g, but the 24th week of pregnancy has been reached. If none of the aforementioned criteria for a live birth or a stillbirth is met, the definition of “miscarriage” or “spontaneous abortion”, *Fehlgeburt*, is used. Unlike live births and stillbirths, miscarriages are not recorded in the German civil register because the law does not legally recognise the aborted foetus as a person. Since May 2013, parents can obtain a certificate from the registry office in response to a petition submitted to the German Bundestag though (German Personal Status Law § 31(3)). Accordingly, there is no obligation for parents to bury deceased babies that weigh less than 500 g; however, collective burials or cremations can be organised by the hospital following the parents’ wish and consent. The distinction between live birth, stillbirth and miscarriage can be also found in the Code of Criminal Procedure (7.04.1987: BGBl. I pp. 1074, 1319; 21.02.2024: Art. 6 BGBl. 2024 I No. 54). Section 90 on “dissecting the body of a newborn” states that the post-mortem examination should focus on determining whether the baby was born alive, and whether he or she was viable to live outside of the uterus.

In Germany, the distinction between stillbirth, live birth and fetal death carries also dramatic implications for the mother in terms of her entitlement to maternity leave and maternal benefits (Sect. 19.2 of the Maternity Protection Act.^2^

For example, to be eligible for maternity benefit in cases of premature birth, neonatal or perinatal death a medical certificate must be submitted along with the birth certificate of the baby to the mother’s health insurance. If the baby was stillborn, weighed less than 500 g or the 24th week of pregnancy was not reached, which is defined as a “an aborted foetus” in medical and legal terms, the mother is not entitled to maternity leave or benefit, nor is she protected against wrongful termination of employment (for further details see the German site of the bereaved parents’ association “Initiative Regenbogen”).^3^ Post-mortem examinations with the consent of the deceased’s next of kin are permitted by law in Germany (Hörnle 2010). However, they are are not regulated by a single binding law within the German federal system (Research Services of the German Bundestag 2016, 3–3000−201/14; Dettmeyer und Madea 2002), nor are neonatal and perinatal autopsies uniformly codified in hospital admission contracts (Dettmeyer und Madea 2004; Hörnle 2010:118–122). Despite this, the Federal Association of German Pathologists compiled a list of medical indications for non-adult post-mortem examination, and a code of conduct (Federal Association of German Pathologists’ Guidelines 2017:4, 11).

In addition, the German Medical Association argues against categorising a corpse as a trivial thing in its statement on “autopsy”, and pleads for the dead body to be handled *lege artis* and with respect in the morgue (2005: p. 24). The German Federal Constitutional Court has ruled that legally, human dignity, and the state obligation to protect human dignity, does not end at death (BVerfGE 30, 173(194); Research Services of the German Bundestag 2018, WD 3–3000−384/18:5; Duttge and Viebahn 2017). In his poignant comment on Article 1 of the German Constitutional Law, which reads “human dignity shall be inviolable”, Herdegen reflected that a corpse does not possess absolute human dignity per se (Herdegen 2005: p. 54); rather, it is imbued with the dignity of the person who once inhabited it. Therefore, strictly speaking, a deceased body deserves respect as it embodies the dignity of the deceased person to whom it once belonged ante mortem. However, in the case of a foetus who died in the womb or a baby who was born dead, neither can attach their dignity to a former living self. Therefore, the question of what ethical respect is due to them arises, as well as whose human dignity they embody.

The Universal Declaration of Human Rights and the Preamble of the Constitution of the WHO address born and living human beings as recipients of dignity, as they are endowed with conscience and reason (Grad 2002). At first glance, it could be argued that foetuses and stillborn babies are excluded from this definition, as they do not meet the aforementioned criteria. This ethical dilemma was resolved in 1994 by the Oviedo Convention on Human Rights and Biomedicine, which sought measures to guarantee the protection of the dignity and identity of “all human beings” without discrimination—in terms of access to healthcare services, biomedical research, and healthcare rights. Given this, such a protection cannot be achieved by relying solely on the possession of dignity and human rights by the individual, but rather by adopting a “protective approach” on the part of state institutions, which are called upon to monitor the protection of human dignity and the rights of their citizens (Beyleveld and Brownsword 2004:-pp. 30–33; Groff and Steger forthcoming).

The German Constitution protects the life of the unborn child as an “embryo” (Article 1.1) and as “human life” (Article 2.2; Research Services of the German Bundestag 2018, WD 3–3000−384/18). This means that foetuses and stillborn babies are legally recognised as having existed in the womb as *nascituri* (German Civil Code, BGBl., 2002 I p. 42, 2909; 2003 I p. 738; 2023 I no. 411, Art. 34.3, §§ 823 ff.). According to the German Embryo Protection Act, the human embryo is granted human dignity from the moment of nidation (1990, BGBl. I p. 2746; 2021 BGBl. I p. 2228, § 8). Therefore, foetuses and stillborn babies can be accorded human dignity, both legally and ethically, and it is the responsibility of a public healthcare system to protect this dignity ante- and post-mortem (Knoepffler 2021: pp. 221 − 23).

Thus, it is not the fundamental inviolability of dignity that should be the focus of all state authority, but rather the creation of a framework of civil law and public health policy that ensures that human dignity remains fully protected. This should also be an ethical standard to be attained in the healthcare system. In the context of a clinical foetal autopsy, in order for the dignity of the foetus to remain inviolable, it requires protection and the creation of ethical health care standards, both in the delivery room during childbirth and later in the morgue. When it comes to the respectful and ethical treatment of the deceased in Germany, the only aspect officially regulated is the respect for the “peace of the dead”, the violation of which is punishable by the law (German Criminal Code, StGB, Sect. 168.9b). In this regard, there are recommendations by the German Hospital Association, religious healthcare institutions, and the Research Services of the German Bundestag referring to the burial and civil status laws of neonatal and perinatal deaths (e.g. Research Services of the German Bundestag 2016, 3–3000−201/14). The Federal Association of German Pathologists published a document recommending a code of conduct for the mortuary and, amongst other indications, a post-mortem examination as standard in cases of stillbirths, miscarriages, neonatal and perinatal deaths (see the “Guidelines of Federal Association of German Pathologists 2017:4, 11; Friemann 2014).

As a result, clinical autopsy following perinatal death is ethically permissible for the benefit of the mother, i.e. for her reproductive and mental health care, during the bereavement process and for future pregnancies (Reed et al. 2021), provided that (1) the dignity of the foetus in the morgue is preserved, i.e. guaranteed *lege artis* by the pathologist, and (2) the autopsy of the foetus, together with the examination of the placenta, can provide significant information to the reproductive medicine team. This ethical recommendation resonates strongly with the original purpose of pathology as displayed in some of the oldest medical universities across Europe reading: *hic locus est ubi mors gaudet succurrere vitae*, “this is the place where death is pleased to aid life”. This Latin motto was the greeting to medical students entering the anatomical theatre, and is still to see in some dissecting rooms today. Consequently, the use of foetal clinical autopsy for the benefit of the mother’s reproductive healthcare does not constitute an unethical instrumentalisation of the foetus. Even though one were to assume that the foetus is being “used” for instrumental purpose, the act of dissecting in itself would not necessarily be considered unethical, as the autopsy does not involve a degradation or a humiliation of the corpse (see e.g. the judgement of the Federal Administrative Court in Bavaria, 21.02.2003:4CS 03.462; Kaufmann 2021).

In addition, the following arguments can also be put forward:

1. Given that the fate of a miscarried or stillborn child cannot be reversed, alas, would it not be even immoral not to use the death of her child to inform the mother’s medical care for future pregnancies and support the mother during the bereavement process?
2. Wouldn’t it be even ethically recommended to implement foetal post-mortem examination as a standard healthcare measure within the limited timeframe of the mother’s fertility?

These hypotheses suggest that in case of an unwanted premature termination of motherhood due to foetal, neonatal or perinatal death, post-mortem examination for reproductive health purposes is morally correct, ethically justified and even ethically recommended (Böcker 2022; Knoepffler 2021).

It is not being argued here that the mother and future siblings demand solidarity of a fotus or a stillborn baby. Instead, a change of perspective is being suggested. Rather than characterising the autopsy of a foetus, stillborn, or neonate as an ethically illegitimate instrumentalisation, it can be viewed as a final act for the sake and wellbeing of others (Knoepffler 2021: pp. 156 − 57;192).

In sum, it can be stated that from an ethical point of view, none of the arguments examined defies the use of foetal clinical autopsy for reproductive healthcare purposes.

## Conclusion

After assessing and discussing the medical, legal, and ethical issues associated with the use of a clinical foetal autopsy for reproductive healthcare in German hospitals, we conclude that it is ethically permissible to perform a post-mortem examination of the foetus, stillborn baby, or neonate for the benefit of the bereaved mother in order to respect her unfulfilled desire to have a child (principle of autonomy). This recommended action is based on the assumptions that, provided that the necessary resources are available (principle of justice) and the dignity of the foetus is protected in the mortuary according to professional standards (*lege artis*), performing a clinical autopsy on the foetus can (1) prevent harm to the mother and the reproductive partners after a diagnosis of infertility disorders, in terms of minimising the risks of a subsequent pregnancy (principle of non-maleficence); and (2) assist in tailoring medical care for the mother in a subsequent pregnancy, thereby increasing the chances of a healthy pregnancy and childbirth (principle of beneficence). There are therefore no ethical arguments against offering and performing a consented clinical autopsy following foetal, neonatal or perinatal death. The clinical autopsy of the foetus, stillborn baby, or neonate serves the medical care and mental health of the mother in the bereavement process and her reproductive healthcare for future pregnancies.

In addition, from an ethical point of view, the offer of a clinical foetal autopsy for reproductive health purposes should be part of the standard clinical examinations provided in German university clinics and hospitals to the bereaved mother and her reproductive partner, in the context of family planning or fertility preservation.

## Data Availability

Not applicable.

## Footnotes

1 E.g. Bundesverband Das frühgeborene Kind: https://www.fruehgeborene.de/index.htm; Bundesverband Verwaiste Eltern und trauernde Geschwister in Deutschland e.V.: https://www.veid.de; Fotografen-Initiative Dein Sternenkind: https://www.dein-sternenkind.eu; Initiative Regenbogen – glücklose Schwangerschaft e.V.: http://initiative-regenbogen.de.

2 https://www.bmfsfj.de/resource/blob/191576/beddabe131e1d1c8e67c55b2c44b73f7/leitfaden-zum-mutterschutz-englisch-data.pdf).

3 http://initiative-regenbogen.de/mutterschutz.html.

## Declarations

### Provenance and peer review

Not commissioned; externally peer reviewed.
